# An examination of the implementation of a patient navigation program to improve breast and cervical cancer screening rates of Chinese immigrant women: a qualitative study

**DOI:** 10.1186/s12905-022-01610-7

**Published:** 2022-02-04

**Authors:** Marquita W. Lewis-Thames, Laura S. Tom, Ivy S. Leung, Anna Yang, Melissa A. Simon

**Affiliations:** 1 Department of Medical Social Science, Center for Community Health, Northwestern University Feinberg School of Medicine, Chicago, IL USA; 2 Department of Obstetrics and Gynecology, Northwestern University Feinberg School of Medicine, Chicago, IL USA; 3 Center for Health Equity Transformation, Northwestern University Feinberg School of Medicine, Chicago, IL USA; 4 Robert H. Lurie Comprehensive Cancer Center, Northwestern University Feinberg School of Medicine, Chicago, IL USA; 5 Departments of Obstetrics and Gynecology, Preventive Medicine and Medical Social Science, Northwestern University Feinberg School of Medicine, 625 N Michigan Ave, Suite 1100, Chicago, IL 60611 USA

**Keywords:** Patient navigation, Health education, Immigrant populations, Chinese American, Minority health

## Abstract

**Background:**

Chinese Americans have lower breast and cervical cancer screening rates than the national average and experience multiple barriers to cancer care. Patient navigators have improved screening and follow-up rates for medically underserved populations, yet investigations of cancer navigation programs and their implementation among Chinese Americans are limited. To address this gap, we used the Consolidated Framework for Implementation Research (CFIR) to examine facilitators and barriers to implementing the Chicago-based Chinatown Patient Navigation Program (CPNP) for breast and cervical cancer screening, follow-up, and treatment.

**Methods:**

Stakeholders from clinical care, supportive care services, and community organizations were invited to participate in qualitative interviews to illuminate implementation processes and stakeholder perspectives of facilitators and barriers to implementing the CPNP. Interviews were audio-recorded, transcribed, and deductively coded according to CFIR domains, including (1) intervention characteristics; (2) outer setting; (3) inner setting; and (4) the implementation process.

**Results:**

We interviewed a convenience sample of 16 stakeholders representing a range of roles in cancer care, including nurses, clinical team members, administrators, physicians, a community-based organization leader, and a CPNP navigator. Findings detail several facilitators to implementing the CPNP, including patient navigators that prepared Chinese-speaking patients for their clinic visits, interpretation services, highly accessible patient navigators, and high-quality flexible services. Barriers to program implementation included limited regular feedback provided to stakeholders regarding their program involvement. Also, early in the program’s implementation there was limited awareness of the CPNP navigators’ roles and responsibilities, insufficient office space for the navigators, and few Chinese language patient resource materials.

**Conclusions:**

These findings provide valuable information on implementation of future patient navigation programs serving Chinese American and other limited-English speaking immigrant populations.

## Introduction

In 2020, national cancer screening rates for female breast and cervical cancer across all races were 72.8% and 81%, respectively [1]. Comparatively, Chinese American women had relatively lower breast and cervical cancer screening rates. For Chinese American women from Chicago’s Chinatown, 60% reported ever receiving a breast cancer screening, and 47% reported ever receiving a cervical cancer screening [2]. In a qualitative study with women from the Chinatown community, barriers to breast cancer screening included “language, time [constraints related to adult children caregiving], not wanting to burden their adult children, and transportation”, while access to navigation services were facilitators to breast cancer screening [3]. Chinese American women with low health literacy and limited English proficiency were 80% less likely to adhere to breast cancer screening guidelines than those with neither limitations [4]. Recognizing these cancer screening disparities and barriers to screening among Chinese American women, investigators from Northwestern University partnered with Mercy Hospital and Medical Center (MHMC) and the Chinese American Service League (CASL), a locally trusted community organization. Guided by the tenets of community-engaged research, this academic-community partnership developed Chicago’s Chinatown Patient Navigation Program (CPNP)—a research program to increase breast and cervical cancer screening rates and timeliness of diagnostic follow-up and treatment for low-income, uninsured, and underinsured Chinese American women residing in Chicago’s Chinatown [5].

For decades, patient navigation programs have significantly reduced delays in breast and cervical cancer screening [6–10]. Patient navigation services are especially effective at connecting racial and ethnic minority groups [6–8], the uninsured [7], and older adults [11] to preventive care. Before the implementation of the CPNP, women of Chinatown reported challenges navigating the healthcare system, including long wait times, difficulty scheduling appointments, and challenges communicating with healthcare professionals [12]. Using strategies adapted from the national Patient Navigation Research Program (PNRP) [9, 13], we launched the CPNP in 2012. The CPNP is a cancer navigation program founded on principles of community-based participatory research, patient-centered care, and shared care strategies. The Shared Care Model is an approach to healthcare delivery grounded on collaborations that bridge the skills and knowledge of interprofessional teams sharing responsibilities and exchanging knowledge and information concerning patient care [14]. Women were eligible for a CPNP navigator if they self-identified as Chinese, were at least 21 years old, and lived in designated Chinatown zip codes. In an evaluation of the first two years of the CPNP, mammography screening rates for residents in neighborhoods with > 20% Chinese ancestry, including neighborhoods like Chinatown, increased from 35.9 to 72.0 per 100 low-income female residents age 50–64 [15]. This analysis demonstrates the effectiveness of the CPNP to engage low-income, non-native English-speaking patients while improving breast screening rates.

The CPNP patient navigators were integrated into the clinical care teams at MHMC and the Chinatown community, thus providing support for clinical services, community wrap-around services, and resource connections [5]. CPNP navigators primarily helped women obtain breast and cervical cancer screenings and, overcome barriers to care. In the event of a cancer diagnosis, navigators assisted patients through treatment and survivorship. In the event of an abnormal screening results, navigation services extended through diagnostic resolution, treatment completion, and surgical consultations [5]. Navigation services were comprehensive, including healthcare navigation, scheduling assistance, interpretation and translation assistance, and distribution of patient education materials. Nine CPNP navigators were hired and trained by CASL and MHMC administrative, nursing, and physician staff. Navigators were hired based on their language skills, familiarity with the Chinatown community and local resources, healthcare and research experience, and compassion to assist patients with multiple barriers to healthcare. Navigators worked in teams of one to five Navigators. Since the inception of the CPNP in 2012, the navigators have engaged over 700 patients and also participated in research activities as members of a Northwestern University research teams. Additional programmatic details about the CPNP and CPNP patient perspectives (from Chinese-speaking adult women in Chicago's Chinatown) are found elsewhere [5, 12, 16, 17].

The success of the CPNP, and similar cancer navigation programs, supports the scaling of patient navigation in cancer care delivery [18, 19]. However, rigorous examination of the adaptation and implementation of patient navigation programs that inform scaling and dissemination efforts remains a gap in the literature. To address this gap, we report the results of a qualitative study using the Consolidated Framework for Implementation Research (CFIR) to examine implementation processes and explore stakeholder perspectives of facilitators and barriers to implementing the CPNP. Results from this qualitative study can begin to inform the uptake and sustainment of future patient navigation programs by providing valuable information on the facilitators and barriers of implementing and disseminating future patient navigation programs serving Chinese Americans and other underserved, limited-English speaking immigrant populations.

## Methods

### Study setting and participants

Chicago’s Chinatown community is home to over 42,000 Chinese immigrants and their families from mainland China, Hong Kong, and Taiwan [20]. Chicago’s Chinatown community is composed of lower-income and working-class Chinese immigrant families [21]. Mercy Hospital and Medical Center (MHMC) is a safety-net hospital located at the periphery of the Greater Chinatown area. MHMC is also the largest provider of screening mammograms and Pap tests for the state-funded Illinois Breast and Cervical Cancer Program (IBCCP). The IBCCP provides eligible uninsured and underinsured Illinois resident women aged 35–64 years with free mammograms, Pap tests, and follow-up diagnostic screenings and services [22]. The Chinese American Service League (CASL) is the largest social service agency in Chinatown. Founded in 1978, CASL’s multilingual professional and support staff offer a variety of social services that support immigrant families.

We recruited stakeholders to participate in interviews; eligible participants were individuals involved in the implementation of the CPNP, including those from clinical care and social service teams from MHMC, staff and social service team members from CASL, and Chinatown community partners. We used departmental rosters and snowball sampling techniques to identify eligible stakeholders. A CPNP navigator and research team member contacted eligible MHMC and CASL staff via telephone calls and emails. Contacted staff were encouraged to help the research team recruit other potential eligible staff from MHMC and CASL via word of mouth. In total, the CPNP coordinator invited 18 potential stakeholders, of which 89% (n = 16) agreed to participate in the interviews.

### Data collection

**Fig. 1:**
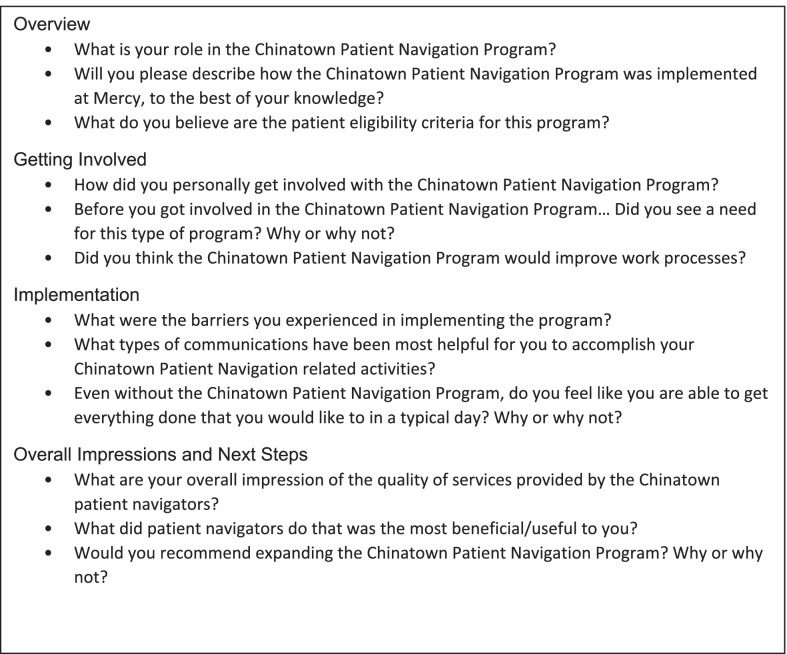
Sample questions from the stakeholder interview guide

Between February–April 2019, we conducted semi-structured interviews with 16 key stakeholders who were involved in the implementation of the CPNP. The interviews were conducted in English via Zoom conference call by two research team members at Northwestern–the project coordinator and a research assistant–independent of the CPNP implementation. Both interviewers were trained by the project principal investigator. Interview questions were guided by the Consolidated Framework for Implementation Research (CFIR) to assess five domains of implementing the navigation program, including the (1) characteristics of an intervention, (2) inner organization setting, (3) outer setting, (4) characteristics of those implementing an intervention, and (5) implementation process [14]. In addition, interview questions probed stakeholders’ perceptions of their roles and knowledge of the program, their overall impressions of the program, and future recommendations for improving the CPNP. Figure 1 details the interview guide. Study staff emailed potential participants the information leaflet and consent form, and all participants provided verbal consent before each interview. Interviews averaged 40 min in length and were audio-recorded with consent from participants. The Northwestern University Institutional Review Board approved all study procedures.

### Data analysis

Audio recordings were transcribed verbatim, and transcripts were verified for accuracy. The interview transcripts were imported into Atlas.ti for analysis. We used a deductive, content analysis approach, with CFIR constructs as the coding framework [23, 24]. Two research team members, uninvolved in the implementation of the CPNP, independently coded the transcripts line-by-line, using the working codebook of categories and concepts drawn from the CFIR constructs. Any differences in coding were discussed to reach a consensus with a third team member. After transcripts were coded, summary tables were created for each CFIR construct, containing summary statements with supporting quotes. Quotes emerged in four major CFIR domains during this process, including (1) intervention characteristics; (2) outer setting; (3) inner setting; (4) the implementation process. We used qualitative descriptions and exemplar quotes to convey the breadth and strength of agreement with a statement, rather than quantifying responses. We also included an interviewee that was a member of the CASL organization as a study team member and co-author (IL). Collectively, her experience, and the prolonged relationship between the principal investigator (MAS), CASL and MHMC enhanced the trustworthiness and credibility of the findings. This study follows the Standards for Reporting Qualitative Research [25].

## Results

We interviewed 16 stakeholders, including MHMC physicians (n = 3), nurses (n = 6), clinical team members (n = 5), a CPNP patient navigator, and a CASL liaison (Table 1). Table 2 details the relevant CFIR domains, constructs, and sub-constructs, as well as the operationalized definitions and exemplar quotes related to each domain.

**Table 1:** Roles of Chinese patient navigation program stakeholders (N = 16)

Participant role
MHMC physicians (n = 3)
Radiation Oncologist
Breast Surgeon
Breast Radiologist
Nurses (n = 6)
Surgery Nurse
Infusion Center Nurse
Nurse Manager
Nurse Practitioner
Breast Cancer Nurse Navigator
Gynecology Nurse Navigator
Clinical Team Members (n = 5)
Mammogram Technician
Oncology Navigator
Oncology Administrator
Senior-level administrator
Oncology Social Worker
Chinatown Patient Navigator Program Navigator (n = 1)
Chinese American Service League Senior-Level Administrator (n = 1)

**Table 2:** Study relevant domain’s constructs, and sub-constructs, definitions and exemplar quotes of the consolidated framework for implementation research

Domain/Construct**/***sub-construct*	Operationalized definition	Exemplar quote
Domain I: Intervention Characteristics	Perception of key stakeholders about the intervention’s external and internal development	
Relative advantage	Perceived advantage of implementing an intervention or program compared to the usual process	The navigators were so good at preparing the patient for what they were going to experience. Even before they got to us. *Nurse*
Complexity	Perceived difficulty of implementing the program	It was not difficult. *Physician*
Design quality and packaging	Stakeholders’ perception of how well the intervention is developed and presented	I think the navigators covered a huge range of patient care services … everything from education regarding insurances, to how family members can get involved in the healthcare system to directly assisting the patient in their diagnostics and then treatment plan completion. *Clinical Team Members*
Evidence strength and quality	Perception of the evidence supporting the intervention and the intervention’s ability to provide the desired outcome	I know that the navigators helped them through, but I don't know, like in detail they would always come to the appointments with the patient and translate and help coordinate their appointments and um, make sure that, you know, they got their treatment and their follow up on time came to their appointments on time. *Physician*
Intervention source	The development of an intervention and how the stakeholders became involved with- or learned about- the program	The first time I met the navigators was when I had a Chinese speaking patients. *Administration*
Domain II: Outer Setting	Describes external influences on implementing the intervention	
Patient needs and resources	How patient needs are adequately addressed by the implementing organization	It's great to be on their wavelength and I have no evidence of this, but I think it helps keep them in the program and get them treated and they're less likely to drift*. Physician*
Domain III: Inner Setting	Identifies the structural characteristics and internal culture that influence implementation	
Implementation climate	An institutions capacity and support for change	
*Relative priority*	Reflects an institution’s shared views on how important an intervention is within the organization relative to their typical workflow(s)	I think the navigator program helped prepare the Chinese patients for the system by introducing them to put up bits and pieces of the system over time. *Administration*
*Goals and feedback*	Development, execution, and communication of the program's goals to the appropriate staff and the stakeholders’ perceptions about the feedback and/or evaluation they received about their involvement in the program	I don't know that there was an expectation of feedback. *Clinical team member*
Networks and communications	Quality and types of communication networks within the implemented program	I don't think they were real involved because the navigators like basically knew what to do when they brought the patients in. *Nurse*
Readiness for implementation	A program’s commitment to a successful implementation	
*Leadership engagement*	The commitment of the program's leadership to implementing the program	They helped give them space to work with and physical, physical space and they were huge proponents of it and proud of it. *Nurse*
*Available resources*	The degree of resources dedicated to implementing the CPNP	I know that they didn't always have resources, but we would come up with something together. *Clinical team member*
Domain IV: implementation Process	Factors related to planning, engaging, reflecting, and evaluating the success of implementing a program	
Engaging	The program’s ability to involve the appropriate talent and strategies to attract participants	The mammographers, the entire cancer doctor team, especially the breast team, um, surgeons and oncologists who are really really involved. Um, I think the other cancer navigators became a big part of it as well as the, um, breast and cervical cancer program, um, employees. *Nurse*
Reflecting and evaluating	Feedback about the progress and quality of implementation; regular debriefing about progress	It would depend on the day. Some days when we were giving a new diagnosis to a patient, it could be, you know, an hour long. Um, if they were coming to look good, feel better and they were sitting with the patient while they were in that program, um, that could easily be an hour long. You know, other days it could be, you know, shorter. So it just really depended on the day. *Clinical team member*

### Facilitators associated with *intervention characteristics (domain I)*

Interviews revealed three relevant CFIR *intervention characteristics* constructs that facilitated the CPNP’s success, (1) relative advantage, (2) complexity, and (3) design quality and packaging.

*Relative Advantage.* Several nurses, for example, felt that “the navigators were so good at preparing the patients…before they got to us” and that the program was “able to help tremendously, [to] have the patients make their appointments and get things done.” A nurse describes that before the CPNP, Chinese-speaking patients did not “really believe or understand what we’re trying to tell them to do”, but with the CPNP support, patients “never experienced that [with] a navigator.” Since the navigators addressed language and cultural barriers, stakeholders reported lower rates of appointment cancellations, better patient outcomes, and patients having an improved understanding of their care plan.

*Complexity.* Stakeholders commented that it was easy to engage in the CPNP. The breast cancer nurse navigator reported that the CPNP navigators “just made it work…they were easy to work with”. An MHMC breast surgeon similarly commented that working with the CPNP navigators was “not difficult.” Nurses had the navigator's cell phone number and office extension, and when patients needed navigation services, “all [they] had to [do] was call them.” For the clinic staff, the navigators were willing “to go to any department” and “do any type of language translation” for their clinical partners. Others commented on the ease of engaging navigators by highlighting that the navigators were timely, readily accessible, and reliable.

*Design quality and packaging.* Stakeholders commented, specifically, on the program’s quality, the interactions with the patient population, and the supplemental patient educational materials. Many stakeholders described the program quality as “outstanding,” “excellent,” and “very successful”, and that the navigators “covered a huge range of patient care services.” Clinical and administrative stakeholders, collectively, reported that the CPNP navigators were easily accessible, received warmly by patients, and consistently demonstrated empathic care. They commented that some patients regarded the navigators as “part of their family” and were even able to contact the navigators “on the weekends and in the evening”. The CPNP navigators provided printed Chinese language patient resource materials about cancer screening and care, that were described as “very good” and “very helpful”.

### Barriers associated with the *intervention characteristics (domain I)*

In addition to the facilitators associated with the *intervention characteristics* domain*,* stakeholder interviews also revealed three barriers associated with this construct related to the (1) complexity, (2) evidence strength and quality, and (3) intervention source construct.

*Complexity.* Several CPNP team members recalled that earlier stages of program implementation seemed to have organizational challenges. The CPNP navigator described that initially, there were times when, “people had no idea what we were, what we were doing. They didn't know how much we could help, how much we understood what's going on […] Most people have never heard of this term [navigators].” Some stakeholders described those difficulties with coordinating work schedules between the CPNP navigators and MHMC’s clinical team members seemed to decrease with time. However, one surgeon expressed that there were a “few instances where someone would not be available or there was no navigator on-site at that time that I needed them. But that was quite rare.”

*Evidence Strength and Quality.* A potential implementation barrier may have been the previous dearth of programs for Chinese-speaking patients at MHMC. For example, a clinical team member noted, “I don’t think there were any other Chinese specific or culturally specific programs [at MHMC].” This lack of exposure to prior existing programs tailored for Chinese patients may have implications on the stakeholders’ readiness to adopt the CPNP and perhaps contributed to some organizational and logistical challenges related to early program implementation.

*Intervention source.* Most MHMC clinical team members reported that they were unfamiliar with the origins and the development of the CPNP. Some noted that their first interaction with the program was when one of their patients arrived with a CPNP navigator, such as one social worker who recalled that “the first time I met the navigators was when I had a Chinese speaking patient.” Other stakeholders (e.g., administrators, clinical team members, CASL’s CPNP liaison) were more knowledgeable about the origins and development of the CPNP, as they served in CPNP leadership roles and were involved in the conceptualization of the CPNP. Interviews revealed that some stakeholders’ limited engagement in the intervention development was related to their hired date. One MHMC breast cancer nurse navigator noted that they didn’t have much of a role in program development, because “it was already established when I assumed the position.” Nevertheless, limited prior awareness and knowledge among some clinical team members about CPNP navigators and their roles and responsibilities potentially contributed to the challenges that CPNP navigators expressed at the onset of the program.

### Facilitators associated with *outer setting (domain II)*

CFIR’s outer setting domain describes external influences on the implementation of the intervention, and the construct of patient needs and resources was noted by stakeholders as a facilitator to intervention implementation.

*Patient Needs and Resources*. The CASL liaison expressed that the motivation for participating in the CPNP was because the program was “useful for our community, [and] for our Chinese patients.” Similarly, several stakeholders recollected that before the CPNP, they believed the program would meet their patients’ needs by addressing language and cultural barriers, guiding patients through the healthcare system, and improving health outcomes. For example, as a social worker acknowledged, “anyone who’s been given a diagnosis of cancer needs to understand—in their own native language—what is going on… I feel that it fits in the patient’s best interest to hear it in their own language.” Others recognized that the CPNP navigators met patient needs by facilitating a “more direct contact to the providers.” Specifically, patients with language barriers were able to receive CPNP services “from the beginning” of their care, at the time of making an appointment, “to the end” of their care visits. For example, a nurse reported that “[I’ll] try to get the job done and move on, whereas the [CPNP] navigators can spend more time really finding out [the patient’s] fears or to give more reassurance [about their care visit]”. When asked, “to what extent stakeholders perceived that the CPNP met patient needs”, several reported improvements in patient outcomes, including increased screening compliance, and a larger volume of Chinese-speaking patients coming in for breast and cervical cancer screenings.

### Facilitators of *inner setting (domain III)*

Key facilitators that emerged from the stakeholder interviews regarding the inner setting construct include: (1) implementation climate, (2) networks and communications, and (3) readiness for implementation.

*Implementation Climate and Sub-Construct Relative Priority.* Many stakeholders praised the CPNP's patient education services, interpretation services, and timeliness to provide supportive care services. The CPNP's navigators provided services that aligned with the MHMC's priorities and complemented the existing workflow. Stakeholders commented that the CPNP navigators were often a patient’s first contact to the healthcare system; thus, navigators were often responsible for orienting and educating patients about their care visits. An administrator noted, for example, that the navigators “helped prepare the Chinese patients for the [healthcare] system.” Also, compared to the CPNP navigators, existing phone-based interpreter services were viewed as more time-consuming, less efficient, and marked by inadequately trained interpreters. As described by one physician, “[With the CPNP navigators] I don’t need this stupid blue phone and I know that [the navigators] understand the situation.” Similarly, a nurse described the hospital’s existing phone-based interpretation services as “frustrating”:

“Trying to schedule a patient for an appointment, that alone might take a couple of hours if I have to go through that translating service, trying to get ahold of the patient, just like phone tag, just making sure that the information is relayed, the prep instructions for certain imaging. That in itself would take half a day and that's just for like one or two patients. Wow. I just wouldn't feel like I would complete the tasks in one day, honestly, if it wasn't without [the navigators] help.”

*Network and Communications.* Communication between clinical team members, program administrators, community partners, and the CPNP navigators included emails, texts, and monthly meetings. One clinical team member described their communication with the navigators as,

“a great relationship. We trusted each other, we trained each other, they trained us, we trained them. It was a good collaboration, you know...it was a very productive relationship […] we were all pretty open and honest with each other…the team would come together and say what they needed.”

Stakeholders also praised the navigators for their responsiveness to resolve emerging problems and their communication skills. For example, a nurse described that the CPNP navigators “knew what to do [and] if they didn't know what to do, they knew who to go to.”

*Readiness for implementation.* In this study, readiness for implementation was assessed via the *leadership engagement* sub-construct. From interviews among the CPNP stakeholders, management-level administrators and physicians acknowledged their roles as CPNP leaders. The CPNP leadership demonstrated their commitment to the program by supporting and engaging with clinical team members, the CPNP navigators, and the Chinatown community. The leadership facilitated the integration of the CPNP into the existing clinical care workflow by connecting navigators with the MHMC oncology service line staff, data support, and IT support. Additionally, the MHMC executive leadership improved CPNP navigators’ work environment. One MHMC nurse reported that executive leadership “helped give [the navigators] space to work”. Moreover, a clinical team member recalled that executive leadership was “very engaged in the community, and especially the Chinatown community”.

### Barriers associates with *inner setting (domain IV)*

Implementation barriers related to CFIR’s inner setting domain are reflected in the following constructs: (1) implementation climate and (2) readiness for implementation.

*Implementation climate and sub-construct goals and feedback*. Several stakeholders, including physicians and nurses, did not recall receiving any feedback from others regarding their participation in the CPNP or additional formal evaluations before the program stakeholder interviews. For example, a nurse navigator mentioned, "I [didn’t] know that there was an expectation of feedback." However, the stakeholders directly involved in the intervention delivery, including the CPNP patient navigator and a nurse, noted various mechanisms such as meetings and other check-ins that enabled “open and honest” feedback.

*Readiness for implementation and sub-construct available resources.* Stakeholders described that early in the program, the CPNP navigators lacked some resources needed to fulfill their responsibilities. As one CPNP navigator recalled, that at the start “we didn't have [an office] space” and had limited Chinese language patient resource materials. Over time, additional supports and resources were provided. As one social worker described, the CPNP navigators “didn’t always have resources [printed in Chinese]” but they recalled that the navigators and social workers partnered to “come up with something together”. The CPNP navigators eventually received a dedicated office space.

### Facilitators associated with the *implementation process (domain IV)*

Interviews among CPNP stakeholders confirmed that the *implementation process* constructs of: (1) engaging and (2) reflecting and evaluating facilitated program implementation.

*Engaging.* The study PI, CPNP navigators, the study staff, and some of the clinicians engaged patients and clinical team members. As reported by a nurse, “the mammographers, the entire cancer doctor team, especially the breast team, surgeons, and oncologists [were] really, really involved” with implementing the CPNP.

*Reflecting and evaluating.* When asked how much time was spent reflecting on or evaluating the CPNP, responses varied. While one nurse recalled, “maybe 15 min daily,” another nurse recalled, “maybe once or twice a month” at the multidisciplinary team conference. However, stakeholders who had more integral CPNP roles spent considerable time on reflections and evaluations. A CPNP navigator expressed that reflecting and evaluating was “something that I do on a daily basis”. For the most part, the frequency of reflection was dependent on the implementer’s role in the program and their interactions with patients.

## Discussion

In this study, we interviewed physicians, nurses, clinical team members, a CPNP patient navigator, and a CASL liaison that participated in the CPNP to gain insight into the facilitators and barriers to implementing a cancer navigation program for Chinese American women in Chicago’s Chinatown. The CFIR framework guided our analysis with four salient constructs (intervention characteristics, inner setting, outer setting, and implementation process). Cancer navigation programs for limited-English speaking women of Chinese ancestry are limited. This is the first study, to our knowledge, that applies an implementation framework to provide important implementation guidance on a cancer navigation program for Chinese American immigrants. Findings revealed stakeholders’ perspectives on the CPNP's capacity to implement a program to reduce language barriers, build productive collaborations among the program’s stakeholders, offer high quality patient navigation services, and improve care outcomes. However, irregular program feedback, limited awareness about the CPNP navigators’ roles and responsibilities, limited office space for the navigators in the early stages of program implementation, and few Chinese language patient resource materials were identified as potential challenges to CPNP implementation. Findings from this qualitative study provide insights to guide the implementation of patient navigation programs and other similar programs, particularly, those that aim to assist non-English speaking patient populations.

Our findings of the utility of navigation services for non-English-speaking patients and medically underserved patients are similar to reports from other patient navigation program studies. Interviews from representatives of county health departments, local clinics, and advocacy organizations participating in a similar patient navigation program noted that navigation services were particularly helpful for patients with limited English proficiency. Furthermore, providing scheduling assistance, interpretation services, and emotional support services were critical to the success of the patient navigation program [26].

This study offers important findings on the barriers to implementing the CPNP, as there are limited investigations of the challenges of implementing patient navigation programs. A review of the implementation of patient navigation programs described that newer programs may lack sufficient resources; however, the review also notes that roles of the navigators tend to evolve to address the immediate care needs of that population, and the availability of resources ultimately becomes available [27]. Stakeholder interviews from this study highlighted that limited office space at the beginning of the program and a dearth of Chinese-language printed patient resource materials posed challenges for CPNP navigators. In turn, CPNP navigators and social workers worked together to mitigate some of the challenges until leadership could garner the necessary resources, such as dedicated office space. The current study also illuminated some programmatic gaps that potentially delayed or hindered program implementation, such as a limited awareness of the CPNP among providers at the program’s inception and missed opportunities for ongoing feedback between clinical team members and the CPNP. It may be helpful for future programs to implement strategies to overcome growing pains when implementing a cancer patient navigation program. We detail lessons learned below.

### Lessons learned from the implementation of a patient navigation program

This study assessed facilitators and barriers for implementing the CPNP. Lessons learned from the stakeholder interviews are summarized into four groups: (1) preparing patients for care; (2) offering continuous patient support; (3) coordinating services well with clinical and administrative partners: and (4) providing high-quality services. These lessons are helpful for identifying practical next steps for implementing similar patient navigation programs. It is important to note that the interpretation service provided by the navigators was a highly useful and complimented service. The linguistically congruent patient navigators provided much needed support in interpretation, and this value was represented in the four summary groups.

#### Patient navigators prepare patients for their care

Providing care visit preparation services may have the potential to strengthen patient navigation services as reflected in relative advantage and implementation climate constructs. CPNP navigators prepared patients for their care visits by assisting with translating pre-clinic visit instructions, scheduling, and debriefing for each patient’s care visit. Navigators that prepared patients for their care had positive implications on care coordination and increased patient adherence to timely health screenings [11, 26]. The current study suggests that patient navigators preparing patients for care visits is an appealing feature of the patient navigation program.

#### Patient navigators offer continuous patient support and care

CFIR constructs involving implementation climate, design quality and packaging, and patient needs and resources highlight the importance of navigators providing continuous and responsive support to strengthen program receptivity among clinical stakeholders. Clinical care team members in this study valued the navigators’ timely response to emergent issues and scheduling of assistance services. Patients valued the continuous support through the care visit, even outside of the scheduled visit. Numerous stakeholders reported on the navigators’ empathic care, including navigating patients through the healthcare system, their accessibility during evenings and weekends, and providing educational materials for the patients and their families.

#### Patient navigators coordinate services well with clinical and administrative partners

We found that complexity, patient needs and resources, implementation climate, networks and communication, and readiness for implementation were constructs related to the care coordination services between navigators and their partners. Stakeholders described that the patient navigators' duties complemented the usual procedures of clinicians and hospital administrators. Activities related to this set of facilitators included providing the needed assistance to clinical partners, providing a novel and needed service (e.g., translational services, following patients from scheduling to follow-up), taking an initiative to resolve emerging challenges, and developing positive working relationships with clinical partners.

#### Patient navigators provide high-quality services

Constructs related to the quality of the offered patient navigation services include relative advantage, complexity, design quality and packaging, patient needs and resources, implementation climate, networks and communications, and readiness for implementation. Stakeholders reported that they valued the services of the patient navigators. Stakeholders described activities demonstrating high-quality services as respecting patients, providing a range of services (e.g., financial support, caregiver support information), improving patient outcomes, and using multiple modes of communication (email, personal cell phone numbers, in-person meetings).

This qualitative study illuminated several potential barriers to CPNP implementation. Below, we summarize the barriers, associated constructs, reported activities that described the barriers, and strategies to mitigate the barriers for implementing a patient navigation program.

#### Limited awareness of the navigators’ roles and responsibilities at the onset of the program

A critical element to initiating a navigation program is that end-users understand the roles and responsibilities of the navigators. This study found that introducing navigators to clinical team members is a potential area for improvement, in that several stakeholders, including oncologists and nurses, reported that their first interaction with a CPNP navigator was when they accompanied a patient. However, other stakeholders, especially those involved in program conceptualization and development, were much more knowledgeable about navigators’ roles and responsibilities and championed within the organization. One potential mitigation strategy to improve the awareness of patient navigation services is to distribute written materials about the navigation program to affiliated partners. Also, opportunities where navigators can provide informational sessions at regular meeting times, such as monthly seminars or lunch and learn meetings, can provide additional exposure to the navigators' roles and responsibilities.

#### Program feedback

We found that most clinical team members did not recall receiving any feedback regarding their participation in the program or any feedback regarding their interactions with the patient navigators (other than their participation in the formal post-intervention stakeholder interviews for this qualitative study). Previous studies encourage the use of regular in-person reviews or opportunities to provide written reviews for valuable ongoing feedback about navigators, their services, and the organization of the newly established program [28]. Ongoing formal feedback between program implementers may be essential for strengthening stakeholder engagement and sustaining program activities.

#### Practice level supports for navigators

Practice-level limitations, involving available resources, was a potential challenge to the implementation of the CPNP. Stakeholders reported frustrations with their existing telephone-based interpreter services, and instead, relied on CPNP navigators to provide interpretations. Another practice-level limitation was the lack of available physical space at the program’s onset. Actions to mitigate these barriers will likely involve clinical and administrative leadership as program champions.

Community-based participatory research principles guided the current study—a strength in the study design. Members of the study team, including the Principal Investigator and the team of CPNP patient navigators actively engaged with CASL and the Chinatown community. Connectivity, a potential outcome of community-engaged research, lends to more honest responses, increased trustworthiness in the researchers, and credibility of the results [29]. We also increased the credibility of the findings through member checking, a qualitative step where the research team summarizes the results and communicates them back to the participants after the data is analyzed. A CPNP navigator contributed to the manuscript writing; thus, enhancing the credibility of the findings and ensuring that the findings represent the true voice of the participants. Another strength of this study is the diverse representation of stakeholders that frequently worked with the CPNP navigators. We reported the unique perspectives of nurses, physicians, other clinical team members, administrators, a CASL community partner, and a CPNP patient navigator on the implementation of the CPNP, thus providing a comprehensive representation of the program.

Given the strengths of this study, there are limitations. First, the data represents a convenience sample of stakeholders who participated in the CPNP. However, of the 18 eligible and invited participants, 16 were interviewed. Thus, we interviewed the majority (89%) of the available and eligible stakeholders. A second limitation is that we only collected the roles and departmental affiliations of each participant and did not collect any sociodemographic information. This study did not provide monetary compensation for participants’ time, identified participants from a highly selective and exclusive participant population, and included study team members that have worked closely with the hospital and the community organization. Therefore, collecting individual participant characteristics may have compromised the anonymity of participants and added additional time/burden in a non-incentivized study. Another limitation is that interviews may be subject to social desirability or recall bias. Last, as this is a qualitative study, we did not assess quantitative cost and differences in health outcomes. These data are useful for understanding the value and implementation of future navigation programs, and further investigation is warranted.

This research adds to the literature on cancer screening patient navigation programs by reporting facilitators and barriers of the CPNP—a patient navigation program adapted for uninsured and underinsured Chinese American immigrant women living in Chicago. Chinese American immigrants are a medically underserved population, and existing programming targeting their care along the cancer continuum is limited. This qualitative analysis revealed facilitators and barriers guided by the CFIR implementation framework. Lessons learned from the reported findings have the potential to improve the implementation of the CPNP and similar programs and inform the development of future navigation programs, especially for medically underserved Chinese American immigrant populations.

## Data Availability

The datasets generated during and/or analyzed during the current study are not publicly available but are available from the corresponding author on reasonable request.
